# Subclavian Vessel Compression Assessed by Duplex Scanning in Patients with Neurogenic Thoracic Outlet Syndrome and No Vascular Signs

**DOI:** 10.3390/diagnostics11010126

**Published:** 2021-01-15

**Authors:** Alban Fouasson-Chailloux, Pierre Menu, Pauline Daley, Giovanni Gautier, Guillaume Gadbled, Pierre Abraham, Marc Dauty

**Affiliations:** 1 CHU Nantes, Service de Médecine Physique et Réadapatation Locomotrice et Respiratoire, 44093 Nantes, France; pierre.menu@chu-nantes.fr (P.M.); pauline.daley@chu-nantes.fr (P.D.); marc.dauty@chu-nantes.fr (M.D.); 2 CHU Nantes, Service de Médecine du Sport, 44093 Nantes, France; 3 Inserm, UMR 1229, RMeS, Regenerative Medicine and Skeleton, Université de Nantes, ONIRIS, F-44042 Nantes, France; 4 CHU Nantes, Explorations Fonctionnelles Vasculaires, 44093 Nantes, France; giovanni.gautier@chu-nantes.fr; 5 CHU Nantes, CCOT, 44093 Nantes, France; guillaume.gadbled@chu-nantes.fr; 6 Vascular and Exercise Investigations, University Hospital of Angers, 49100 Angers, France; piabraham@chu-angers.fr; 7 Vascular Medicine, University Hospital of Angers, 49100 Angers, France; 8 Mitovasc, UMR CNRS 6015 INSERM 1083, LUNAM University, 49100 Angers, France

**Keywords:** neurogenic thoracic outlet syndrome, bilateral, duplex imaging, vascular, rehabilitation

## Abstract

Neurogenic thoracic outlet syndrome (NTOS) is the most frequent form of TOS. It may affect both sides, but specific complementary exams are lacking. We aimed to evaluate duplex scanning results in a group of patients with unilateral or bilateral NTOS and no clinical vascular signs, referred for rehabilitation. We performed a retrospective observational study in patients with unilateral or bilateral NTOS and no vascular symptoms. Subclavian vessels were assessed by duplex scanning. Compressions were considered in case of >50% of increased or decreased blood flow. A total of 101 patients met NTOS criteria; mean age was 40 +/− 10.2; 79.2% women. Seventy patients had a unilateral NTOS and 31 a bilateral form. Duplex scanning showed that 56.4% of the patients had vessels compression, 55.7% in the unilateral group and 58.1% in the bilateral (*p* = 0.81). In unilateral NTOS, 21 (30%) patients had bilateral vascular compression, 17 (24.3%) had ipsilateral compression and 1 (1.4%) had contralateral compression. In bilateral NTOS, 15 (48.4%) had bilateral compression and 3 (9.7%) compression on only one side. We found a significant difference of the rate of vascular compressions between symptomatic and non-symptomatic upper-limbs, 54.5% vs. 32.9%, respectively, (*p* = 0.002) and a significant association between symptomatic upper-limbs and vascular compression (OR = 2.45 [95%IC: 1.33–4.49]; *p* = 0.002). The sensitivity and the specificity of the duplex scanning were 54.5% and 67%, respectively. The ROC curve area was of 0.608 [95%IC: 0.527–0.690]. Despite a highly significant association between symptomatic upper-limbs and vascular compression, duplex scanning did not help make the diagnosis of NTOS.

## 1. Introduction

Thoracic outlet syndrome (TOS) includes all manifestations due to the compression of the neurovascular structures of the thoracic inlet [1]. Its frequency is estimated from 0.3 to 8% of the population [2,3]. Clinical forms are generally distinguished according to the structures involved: neurogenic TOS (NTOS), venous TOS and arterial TOS [3,4]. NTOS is the most frequent form and represents about 90% of cases [1,3,5]. It usually associates anatomic predispositions and various local factors which may lead to intermittent compression of the brachial plexus at the supraclavicular scalene triangle and/or at the sub-coracoid space levels [5,6,7]. NTOS is a chronic and painful condition responsible for upper-limb functional disability [6,8]. It may affect both sides in 23–58% of cases [9,10]. Women are more frequently affected in about 70% of the cases, at a mean age of 40 [6,11]. Conservative management associating physiotherapy and rehabilitation programs is the initial treatment of choice for NTOS with good results [12,13,14]; it includes posture exercises, muscular strengthening and stretching [3,5]. Surgical procedures should be proposed to patients in case of conservative treatment failure [6,15]. Because of the lack of specificity of NTOS, especially concerning complementary exams, clinical guidelines have been recently proposed so as to be more consensual diagnosis [4,6,12,16,17,18]. Yet, some studies have assessed the interest of duplex imaging in NTOS [19,20], which may seem relevant given the anatomical proximity between subclavian vessels and brachial plexus. Interestingly, these surgical studies showed that patients had vascular compressions in about 50% of cases. However, these studies included only patients that were to undergo surgery, because they did not respond favorably to the conservative treatment. These studies seem to have evaluated only NTOS with unilateral symptoms [19,20]. Moreover, both these studies exclusively assessed NTOS patients with associated clinical signs interpreted as vascular compression.

In this study, due to the anatomical link between subclavian vessels and brachial plexus, we aimed to evaluate retrospectively duplex scanning results in a group of patients with NTOS and no clinical vascular signs, referred for an evaluation prior to a rehabilitation program. Furthermore, we also aimed to compare results between patients with unilateral and bilateral NTOS.

## 2. Materials and Methods

### 2.1. Population

**Figure 1:**
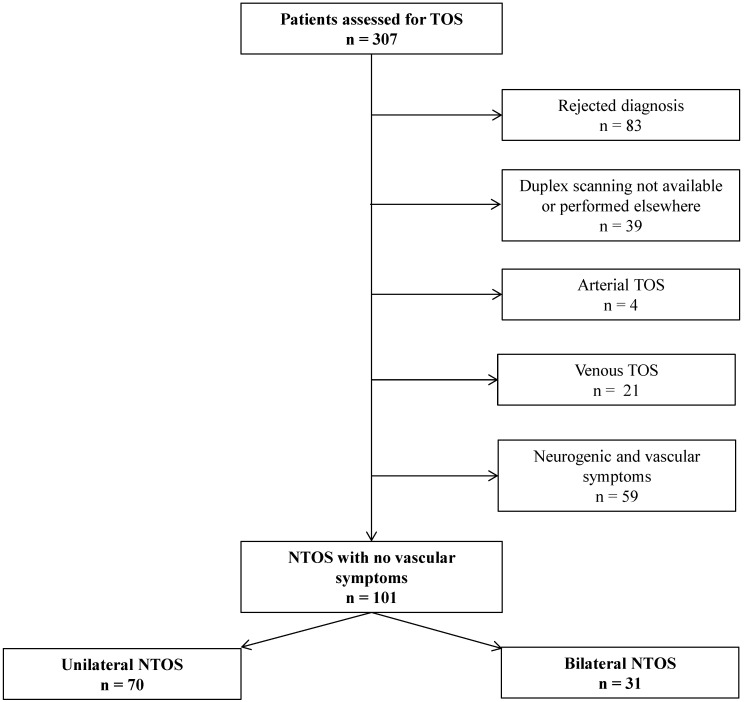
Flow-chart of the patients’ inclusion in the study.

Since 2015, we propose a specific consultation dedicated to TOS in case of ineffective physiotherapy in order to consider intensive physiotherapy program in our rehabilitation center. Patients are usually referred by upper-limb surgeons (vascular surgeons or orthopedists), rheumatologists or vascular physicians. For the past 3 years (2017, 2018, 2019), 307 patients have been evaluated. All the patients had previously a duplex scanning performed in the vascular department of the University Hospital, cervical X-rays and a cervical MRI. After analyzing patients’ medical records, we included retrospectively 101 patients with NTOS. To be included in the study, patients had to fulfill diagnostic criteria for unilateral or bilateral NTOS according to the Consortium for Research and Education on thoracic outlet syndrome (CORE-TOS) [6,12,18]. Patients were excluded in case of other potential diagnosis (cervical radiculopathy for example) or previous TOS surgery, if duplex scanning was unavailable or performed in another center, in case of vascular TOS (arterial or venous) or if patients had both symptoms of NTOS and vascular involvement. Figure 1 presents flow-chart of the patients’ inclusion in the study.

### 2.2. Neurogenic Thoracic Outlet Syndrome Diagnosis

All the patients were examined by two experienced physicians specializing in the rehabilitation of TOS. Clinical description of the NTOS patients according to CORE-TOS criteria is presented in Table 1. In case of associated vascular symptoms or clinical signs, patients were excluded: swelling, discoloration, Raynaud syndrome, sub-clavicular bruit, dynamic pulse abolition or distal ulceration [4]. Indeed, we aimed to eliminate potential neuro-vascular forms.

### 2.3. Duplex Scanning

Duplex scans were performed with a Philips HD15 Ultrasound system, KPI healthcare Inc., Neuss, Germany. All the exams were realized by experienced vascular sonographists from the Vascular Medicine Department of the University Hospital with a standardized protocol. The duplex scans assessed subclavian vein and artery caliber, and blood flows at rest, lying down and in the sitting position with the head turned to the contralateral side with a shoulder retropulsion of 30°, at 90° and 120° of abduction. Compressions were considered in case of more than 50% increase or decrease blood flow in subclavian vessels, either in the artery or in the vein, or on in both [21,22].

### 2.4. Ethics

Applicable institutional and governmental regulations concerning ethics were followed during the course of this study. The data report form was declared to the French data protection authority (CNIL) and to the Research Department of the University Hospital. Since data were collected retrospectively and that patients’ management was not modified, according to French law, this study did not need to be approved by a research ethics committee and no patient informed consent was required (articles L.1121-1 paragraph 1 and R1121-2, Public Health code).

### 2.5. Statistics

Statistical analysis was performed using the SPSS 23.0^®^ software package (IBM corp., Dublin, Ireland). Quantitative variables are given in mean values and standard-error (SD) and qualitative variables are given in numbers and frequency. Univariate analysis (independent *t*-test after variances analysis by Levene’s test) and χ^2^ test were used to compare quantitative and qualitative parameters, respectively. As some patients had bilateral NTOS, we performed a statistical analysis taking the upper-limbs as reference, using a *t*-test, and Odds Ratio (OR) was calculated for duplex scanning positivity depending on the symptomatic or non-symptomatic upper-limb. The interest of duplex scanning to diagnose NTOS was assessed using sensitivity, specificity, likelihood ratios and receiver operating characteristic (ROC) curve area. The positive likelihood ratio allows to consider the diagnosis if it is superior to 10 and the negative likelihood ratio allows to reject the diagnosis if it is inferior to 0.10 [23]. The ROC curve area was interpreted as excellent (>0.9), good (0.8–0.9), fair (0.7–0.8), poor (0.6–0.7) or failed (0.5–0.6) [24].

The results were considered significant at the 5% critical level (*p* < 0.05).

## 3. Results

We finally included 101 patients who met NTOS diagnosis criteria without symptoms of vascular compromise (Figure 1); their mean age was 40 +/− 10.2, of which 79.2% were women. The mean symptoms duration was 2.9 +/− 2.0 years. A history of upper-limb limb injury was reported in 16.8% of the cases and a radiologic abnormality was found in 12.9% (mega-apophysis of C7 and cervical rib). 

The patients fulfilled diagnostic criteria for unilateral or bilateral NTOS according to the CORE-TOS criteria (Table 1). 100% of the patients were complaining of pain in the neck, shoulder, arm, and/or hand; 88.1% had numbness, paresthesia and or weakness in the arm, hand or digits; 99% had pain, paresthesia and/or weakness exacerbated by elevated positions and 84.2% exacerbated by repetitive arm/hand use; 100% had local tenderness on palpation over the scalene triangle and/or sub-coracoid space; 75.2% had arm, hand and/or digit paresthesia on palpation over the scalene triangle and/or sub-coracoid space.

Seventy patients had a unilateral NTOS (69.3%) and 31 a bilateral form (30.7%). No difference was found between the two sub-groups of patients (Table 2).

Duplex scanning showed that 56.4% of the patients had vessels compression, 55.7% in the group with unilateral TOS and 58.1% in the group with bilateral TOS (*p* = 0.81) (Table 3). 33.3% of the compressions were arterial, 31.6% were venous and 35.1% were arterial and venous. In the patients with unilateral symptoms, 21 (30%) had bilateral vascular compression, 17 (24.3%) had ipsilateral compression and 1 (1.4%) had contralateral compression. In the patients with bilateral symptoms, 15 (48.4%) had bilateral compression and 3 (9.7%) compression on only one side (Table 3). Taking upper-limbs as units, we found a significant difference of the rate of vascular compression between symptomatic upper-limbs and non-symptomatic upper-limbs, 54.5% vs. 32.9% respectively (*p* = 0.002) (Table 4). We also found a significant association between symptomatic upper-limbs and vascular compression on duplex scanning (OR = 2.45 [95%IC: 1.33–4.49]; *p* = 0.002). The sensitivity and the specificity of the duplex scanning were 54.5% [95%IC: 46–62%] and 67% [95%IC: 55–77%], respectively. The positive likelihood ratio was 1.65 [95%IC: 1.14–2.40] and the negative likelihood ratio was 0.68 [95%IC: 0.52–0.86]. The ROC curve area was poor to failed, at 0.608 [95%IC: 0.527–0.690].

## 4. Discussion

NTOS is a challenging diagnosis due to the heterogeneity of the symptoms and the lack of specific laboratory exams [1,3,4,16]. Recently, guidelines have been proposed to clarify and help make the diagnosis which is based on the absence of other probable diagnosis [18]. On the contrary, vascular forms of TOS are easier to explore and duplex scanning appears as a sensitive and specific tool to explore non-invasively arterial and venous TOS [4,25,26]. Duplex ultrasound has a high sensitivity (78–100%) and a high specificity (82–100%) in the diagnosis of VTOS [27,28]. Some authors have evaluated the use of vascular scanning in NTOS, especially before surgery. Indeed, brachial plexus and subclavian vessels are close [19]; so, showing a vascular compression could be an indirect sign of nerves impingement, even if this opinion is debated [1]. Molina et al. assessed systematically, 148 consecutive patients with unilateral NTOS prior to arterial decompression [19]. Yet, all their patients presented clinical signs that could have evoked arterial impingement, especially pallor of the hands at 180° of elevation, and were qualified by the authors of neurogenic-arterial NTOS. Duplex scanning found that 51% of the patients had arterial pinching or obstruction during abduction and 8% had bilateral compression; 7.4% had venous compression. Likes et al. also evaluated the coexistence of arterial compression and NTOS in a pre-surgical series [21]. Of 423 patients, who had undergone surgical intervention, they assessed (with duplex scanning) 22 patients with NTOS and associated signs of arterial compromise. They reported 12 patients (54.5%) with significant compression of the subclavian artery. Recently, Orlando et al. retrospectively reported a series of 143 surgical unilateral NTOS that had venous and arterial duplex scanning before surgery. The patients seemed to have clinical signs of vascular impingement: discoloration, swelling or positive Adson test. They found that 49% of the patients had vascular compression, 31% had ipsilateral compression, 8% had contralateral compression and 10% had bilateral compression. These studies took into consideration only pre-surgical NTOS associated to “vascular signs”, so we chose to specifically study patients without these signs of arterial or venous compromise in a population of non-surgical NTOS, to evaluate the interest of duplex scanning in these patients. Indeed, despite the absence of clinical signs evocating vascular compression, we found similar results with 56.4% of compression in our population: 55.7% in unilateral NTOS and 58.1% in bilateral forms. The first explanation for these findings could be the fact that vascular compression may be an indirect sign of nerves impingement due to the anatomical proximity between these structures. However, we can evoke that clinical arguments of vascular impingement used in studies, have usually poor sensitivity and specificity. Indeed, even if discoloration, swelling or a positive Adson test are noticed, duplex scanning showed no compression in about half of the cases [19,20,21]. One explanation could be that Adson test positivity is not necessarily due to a symptomatic vascular compression. Its interest seems modest in the diagnosis of NTOS with a high variability of positivity from 22 to 100%, and significant rates of false positive tests in a healthy population, from 9 to 53% [1,29,30]. Another explanation could be that color changes and coldness, frequently reported in NTOS might be due to an over-activity of the sympathetic nervous system, whose fibers are next to the C8 and T1 nerve roots [1]. In case of plexus irritation, sympathetic fibers could be activated, inducing Raynaud’s phenomena. Anyway, excluding vascular signs in our study had a low impact on the number of positive duplex imaging, which seems to give this imaging a low contribution to help make the diagnosis of NTOS with vascular sign or not.

The use of duplex scanning in NTOS has also been debated because even our results and those of previous studies found 49–56.4% of positivity [19,20,21]; it has been shown that in healthy volunteers, vascular compressions are also noticed from 12 to more than 20% of the cases [22,31,32]. In NTOS patients, Orlando et al. [20] found 18% of patients with duplex compression on the asymptomatic upper-limb whereas in our study, this rate was higher (31.4%). Yet, one of the main originality aspects of our work is the high number of bilateral NTOS (30.7%)—no cases in Orlando et al. and Molina et al. studies [19,20], and only three in those of Like et al. [21]. This enabled us to show that out of the 18 bilateral NTOS patients with vascular compressions, 15 had bilateral compression (83%). Considering symptomatic sides vs. non-symptomatic ones, we showed that upper-limbs with NTOS had a 2.45 times higher rate of vessels compression than non-symptomatic side in patients with NTOS (OR = 2.45 [95%IC: 1.33–4.49]; *p* = 0.002). Yet, in our population, the high association between NTOS and vascular impingement did not help in making or rejecting the diagnosis. Indeed, vascular compression with duplex scanning had poor sensitivity, specificity and likelihood ratio. Thus, we could consider that in this context the realization of duplex scanning had poor interest.

Our study has limitations due to its retrospective design. Indeed, we cannot totally exclude some errors in the data collection or in the transcription of the clinical examination. Yet, we have limited these types of errors by using a standardized data collection and clinical examination based on the CORE-TOS criteria. Furthermore, our study is only descriptive; we found an association between NTOS and compression in duplex scanning, but no causal link could be clearly established between imaging findings and clinical signs. We assessed sensitivity, specificity and likelihood ratios using non-symptomatic upper-limbs as controls, which could be debated. Hence, a new prospective study, comparing patients with NTOS to healthy subjects, could be proposed to confirm these results. However, to our knowledge, our study is the 1st which assessed duplex compression of subclavian vessels in a non-surgical population of NTOS with a high rate of bilateral forms.

## 5. Conclusions

In the absence of clinical signs usually considered evocative of vascular compression, we found a high rate of subclavian vessel compression with duplex scanning in patients with NTOS. No difference was found between patients with unilateral or bilateral symptoms. However, despite a highly significant association between symptomatic upper-limbs and vascular compression, duplex scanning did not help make the diagnosis of NTOS because of poor sensitivity and specificity.

## Figures and Tables

**Table 1:** Patients with clinical diagnosis criteria for neurogenic thoracic outlet syndrome (NTOS) according to Consortium for Research and Education on thoracic outlet syndrome [6,18].

Diagnosis Criteria for NTOS	n (%)
No other probable diagnosis	101 (100%)
Symptoms duration ≥ 12 weeks	101 (100%)
Principal symptoms	
1a: Pain in the neck, upper back, shoulder, arm, and/or hand.	101 (100%)
1b: Numbness, paresthesia, and/or weakness in the arm, hand, or digits.	89 (88.1%)
Symptom characteristics	
2a: Pain/paresthesia/weakness exacerbated by elevated arm positions.	100 (99.0%)
2b: Pain/paresthesia/weakness exacerbated by prolonged or repetitive arm/hand use.	85 (84.2%)
2c: Pain/paresthesia radiate down the arm from the supraclavicular or infra clavicular spaces.	75 (74.3%)
Clinical History	
3a: Symptoms began after occupational, recreational, or accidental injury of the head, neck, or upper extremity, including repetitive upper extremity strain or overuse.	67 (66.3%)
3b: Previous ipsilateral clavicle or first rib fracture, or known cervical rib.	3 (2.9%)
3c: Previous cervical spine or ipsilateral peripheral nerve surgery without sustained improvement in symptoms.	17 (16.8%)
3d: Previous conservative or surgical treatment for ipsilateral TOS.	0 (0.0%)
Physical examination	
4a: Local tenderness on palpation over the scalene triangle and/or sub-coracoid space.	101 (100%)
4b: Arm/hand/digit paresthesia on palpation over the scalene triangle and/or sub-coracoid space.	76 (75.2%)
4c: Objectively weak handgrip, intrinsic muscles, or digit 5, or thenar/hypothenar atrophy.	0 (0%)
Provocative maneuvers	
5a: Positive upper limb tension test (ULTT).	90 (89.1%)
5b: Positive 3-min elevated arm stress test (EAST).	98 (97.0%)

**Table 2:** Population with NTOS and comparison between patients with unilateral and bilateral forms.

	NTOS (n = 101)	Unilateral (n = 70)	Bilateral (n = 31)	*p*
Female/Male/	80/21	56/14	24/7	0.79
Age, years +/− SD	40.0 +/− 10.2	40.1+/− 10.6	40.0 +/− 9.4	0.99
Weight, kg +/− SD	68.0 +/− 14.9	67.5 +/− 15.4	69.2 +/− 13.8	0.59
Height, cm +/− SD	165.7 +/− 7.5	165.1 +/− 7.3	167.0 +/− 7.8	0.21
Body mass index, kg/m^2^ +/− SD	24.8 +/− 5.4	24.8 +/−- 5.8	24.8 +/− 4.6	0.98
Symptoms duration, years +/− SD	2.9 +/− 2.0	2.8 +/− 2.0	3.1 +/− 2.4	0.58
Head/Neck/Shoulder accidental injury, n (%)	17 (16.8%)	12 (17.1%)	5 (16.1%)	0.99
Radiographic abnormalities n (%): - Elongated C7 transverse process - Cervical rib	13 (12.9%) 11 (10.9%) 2 (2.0%)	9 (12.9%) 8 (11.4%) 1 (1.5%)	4 (12.9%) 3 (9.7%) 1 (3.2%)	0.87 ^a^
Positive electromyography	17 (16.8%)	12 (17.1%)	5 (16.1%)	0.99

NTOS = neurogenic thoracic outlet syndrome; SD = standard deviation. SD = standard deviation. ^a^ = χ2 test.

**Table 3:** Vessels assessment by duplex ultrasound scanning in patients with unilateral and bilateral NTOS.

Duplex Scanning Results	NTOS (n = 101)	Unilateral (n = 70)	Bilateral (n = 31)	*p*
No compression n (%)	44 (43.6%)	31 (44.3%)	13 (41.9%)	0.81 ^a^
Compression n (%)	57 (56.4%)	39 (55.7%)	18 (58.1%)	
- Arterial - Venous - Arterial and venous	19 18 20	11 14 14	8 4 6	
Ipsilateral compression	20 (19.8%)	17 (24.3%)	3 ^b^ (9.7%)	
Contralateral compression	1 (1.0%)	1 (1.4%)	N/A	
Bilateral compression	36 (35.6%)	21 (30.0%)	15 (48.4%)	

NTOS = neurogenic thoracic outlet syndrome. ^a^ = χ2 test; ^b^ both sides are symptomatic but only one showed vessels compression.

**Table 4:** Vessels assessment by duplex ultrasound scanning depending on the symptomatic or non-symptomatic upper-limb.

Duplex Scanning Results	Symptomatic Upper-Limbs (n = 132)	Non-Symptomatic Upper-Limbs (n = 70)	*p*
No compression n (%)	60 (45.5%)	47 (67.1%)	0.002
Compression n (%)	72 (54.5%)	23 (32.9%)	

## Data Availability

The data presented in this study are available on request from the corresponding author. The data are not publicly available due to ethical reasons.
